# Supplementary material for: Plasma Metabolites Associate with All-Cause Mortality in Individuals with Type 2 Diabetes
Source: Metabolites. 2020 Jul 31;10(8):315. doi: 10.3390/metabo10080315 (PMC7464745; doi:10.3390/metabo10080315)
Supplement: Supplementary file 1 [file metabolites-10-00315-s001.zip › metabolites-869993-Supplementary/Suplementary.pdf]

Material S1.

Endpoint definitions and biochemical measurements

Myocardial infarction was defined on the basis of International Classification of Diseases, ninth revision (ICD-9) code 410 or ICD-10 code I21. Fatal and non-fatal stroke was defined using codes 430, 431, 434 and 436 (ICD-9) and I60, I61, I63, and I64 (ICD-10). Cigarette smoking was elicited by a self-administered questionnaire, with current cigarette smoking defined as any use within the past year. Measurements of fasting total cholesterol, HDL cholesterol, triglycerides, glucose, insulin and hba1c were made according to standard procedures at the Department of Clinical Chemistry at Malmö University Hospital. LDL cholesterol was estimated with the Friedewald equation. HOMA-IR was calculated as the fasting insulin concentration (mU/L) multiplied by the fasting glucose concentration (mmol/L) divided by 22.5.

Material S2.

Normalization

Normalization was performed using metabolite measurements in the quality control samples. First, a low-order nonlinear locally estimated smoothing function was fitted to the metabolite signals in the quality control samples as a function of the injection order. The α-parameter, reflecting the proportion of samples to be used when constructing the correction curve, was set to 2/3. Using this function, a correction curve for the analytical samples was interpolated, to which the metabolite measurements in the analytical samples were normalized. The normalization was performed in R 3.6.0.
