# Supplementary material for: Plasma Metabolites Associate with All-Cause Mortality in Individuals with Type 2 Diabetes
Source: Metabolites. 2020 Jul 31;10(8):315. doi: 10.3390/metabo10080315 (PMC7464745; doi:10.3390/metabo10080315)
Supplement: Supplementary file 1 [file metabolites-10-00315-s001.zip › metabolites-869993-supplementary/Supplementary Figures.pdf]

Supplementary Figure S1. Study design of the Malmö Diet and Cancer – Cardiovascular Cohort (MDC-CC) and the Malmö Preventive Project (MPP).

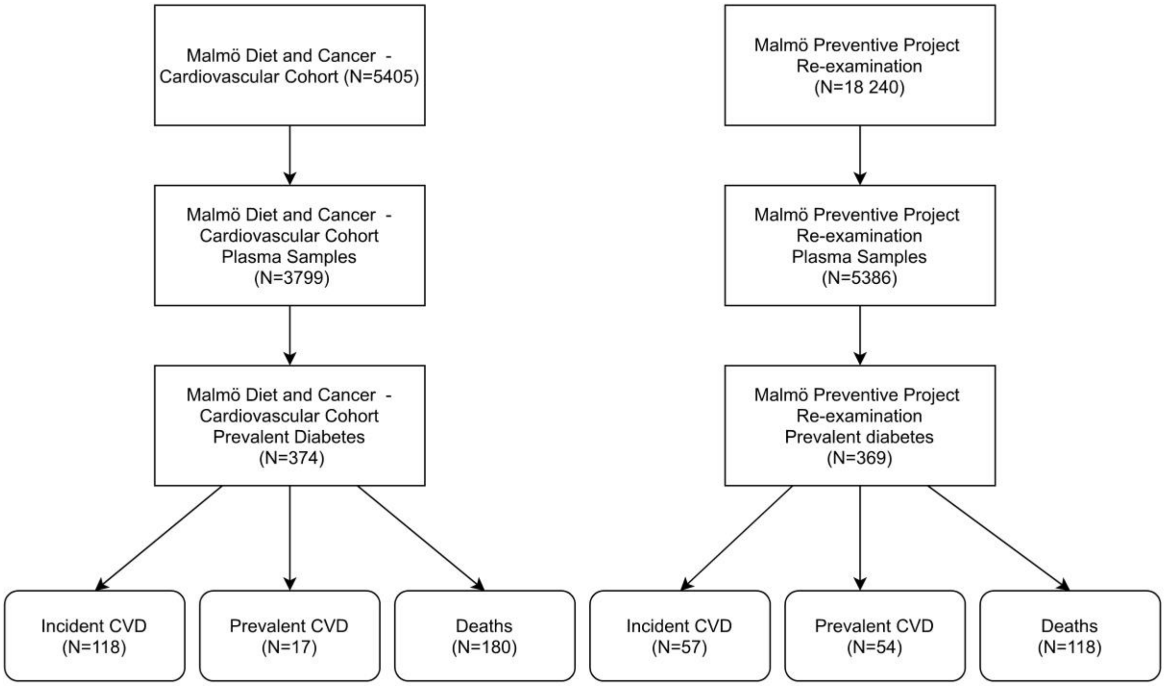

Supplementary Figure S2. Annotation of Acisoga.

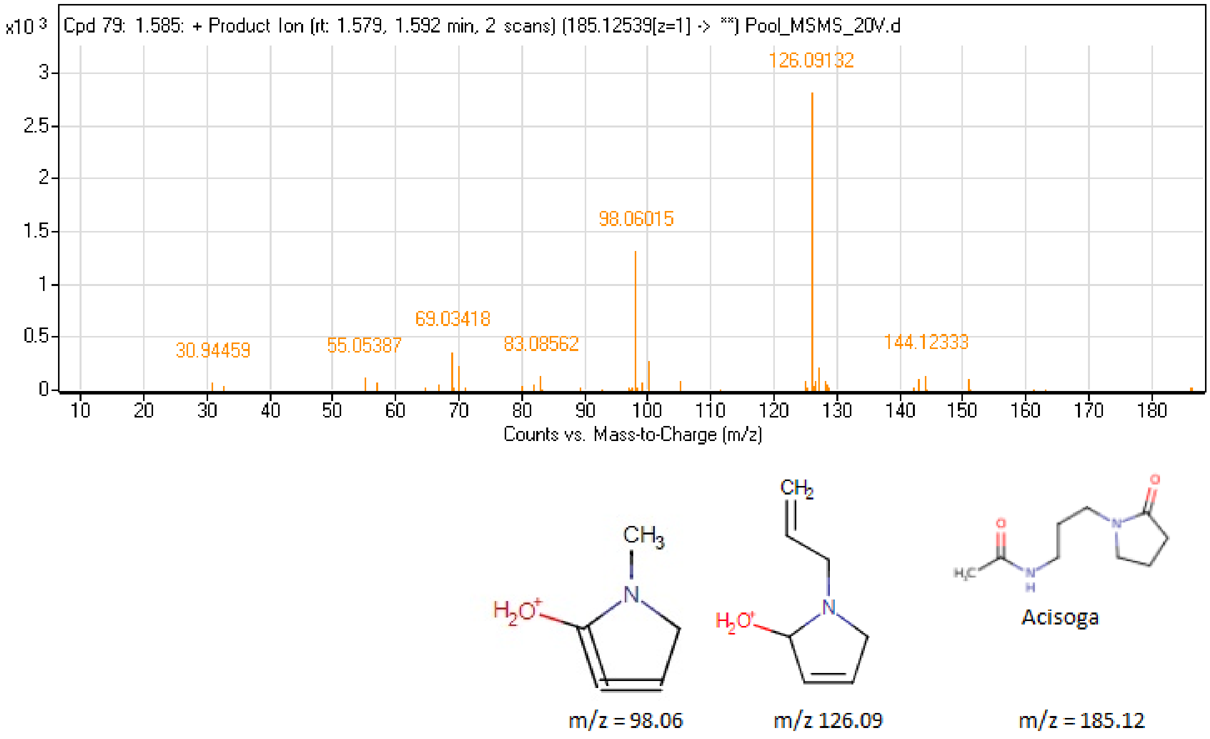

Annotation of Acisoga by MS/MS-analysis using 20V collision energy and isolation width of 1.3 m/z. Putative molecular fragments are indicated.

Supplementary Figure S3. Annotation of Acylcarnitine C10:3

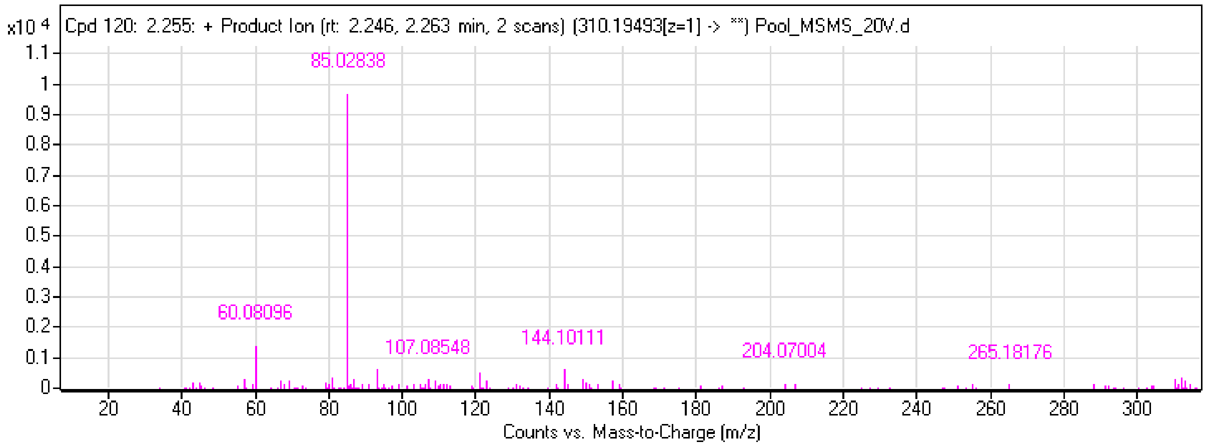

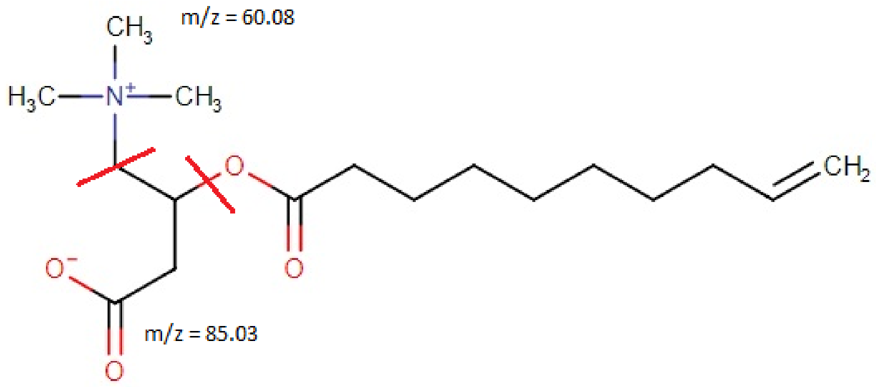

Annotation of C10:3 Acylcarnitine by MS/MS-analysis using 20V collision energy and isolation width of 1.3 m/z. Putative molecular fragments are indicated.

Supplementary Figure S4. Annotation of 1-methyladenosine

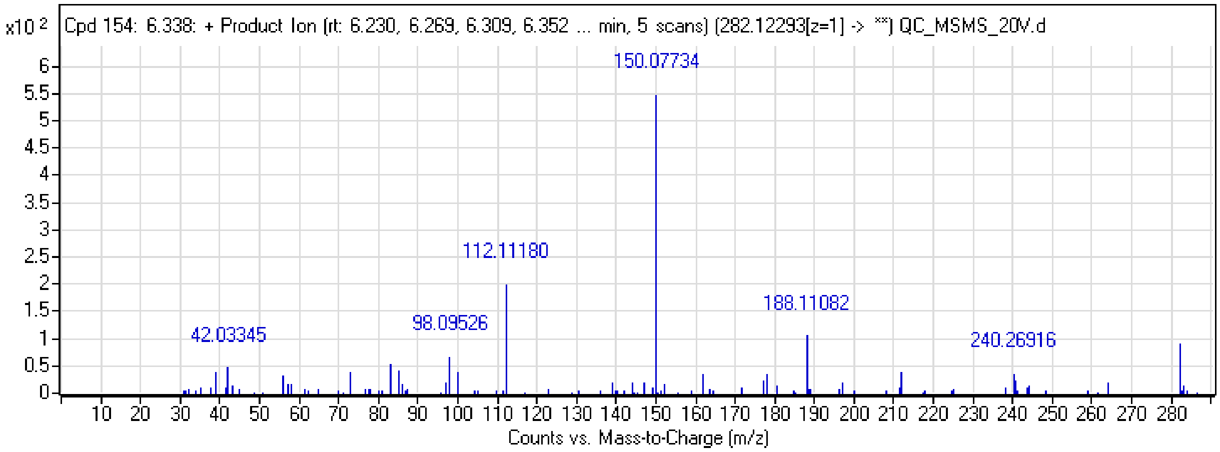

Annotation of 1-methyladenosine by MS/MS-analysis using 20V collision energy and isolation width of 1.3 m/z. A putative identity of 1-methyladenosine was confirmed by mathcing m/z and fragmentation spectra with spectra from HMDB (HMDB03331) and Metlin (6888).

Supplementary Figure S5. Annotation of Dimethylguanidino valerate (DMGV)

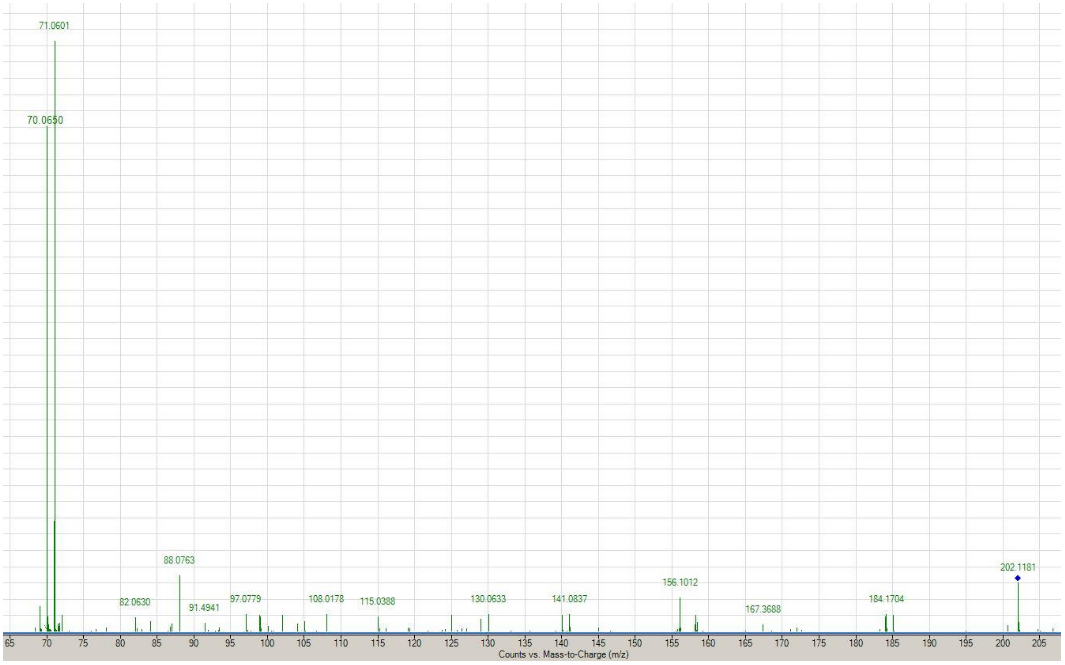

Annotation of dimethylguanidino valerate (DMGV) by MS/MS-analysis using 20V collision energy and isolation width of 1.3 m/z. The identity of DMGV was confirmed previously [1] by mathcing accurate mass and fragmentation spectra of m/z 202.1192 with previously published spectra of DMGV [2]. Additional evidence for correct identification of DMGV was provided by conducting a genome-wide association study of DMGV, confirming the variants in the *AGXT2* gene to be associated with DMGV levels[1].

Supplementary Figure S6. Annotation of Urobilin

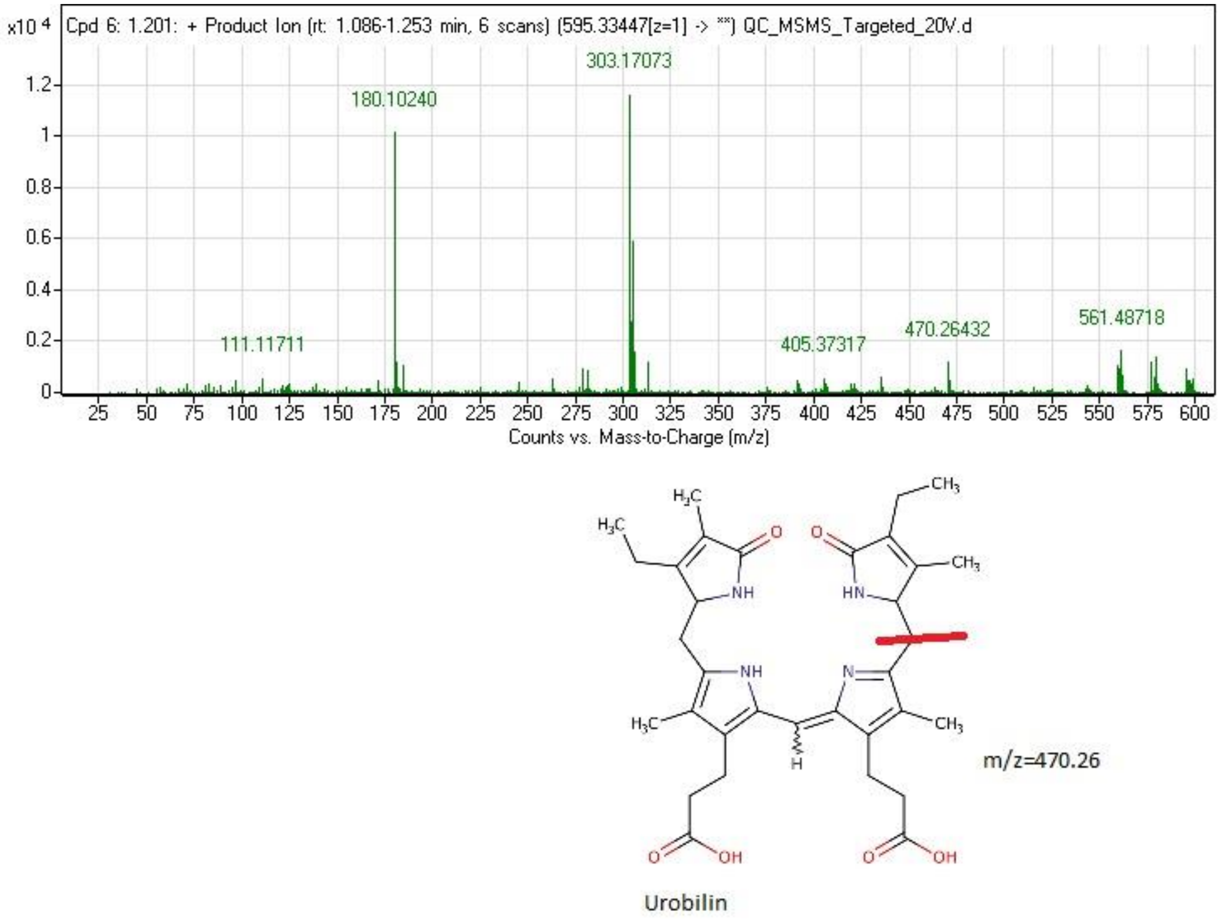

Annotation of Urobilin by MS/MS-analysis using 20V collision energy and isolation width of 1.3 m/z. Putative molecular fragments are indicated.

Supplementary Figure S7. Annotation of N2,N2-dimethylguanosine

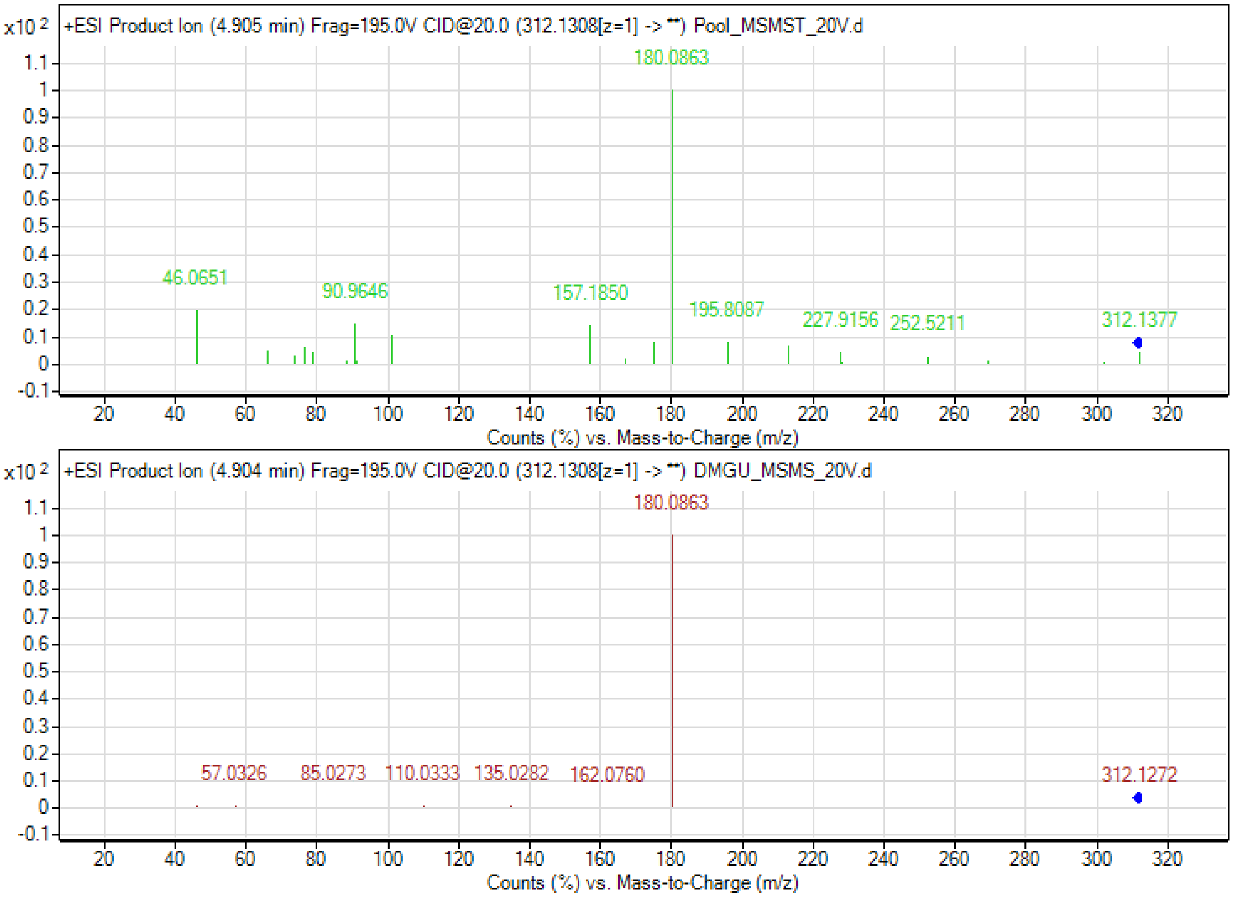

Annotation of N2,N2-dimethylguanosine by MS/MS-analysis using 20V collision energy and isolation width of 1.3 m/z. m/z 312.13 was isolated and fragmented in pooled plasma sample (upper panel) and matched with corresponding fragmentation spectra for N2,N2-dimethylguanosine standard (lower panel).

Supplementary Figure S8. Annotation of Hippurate

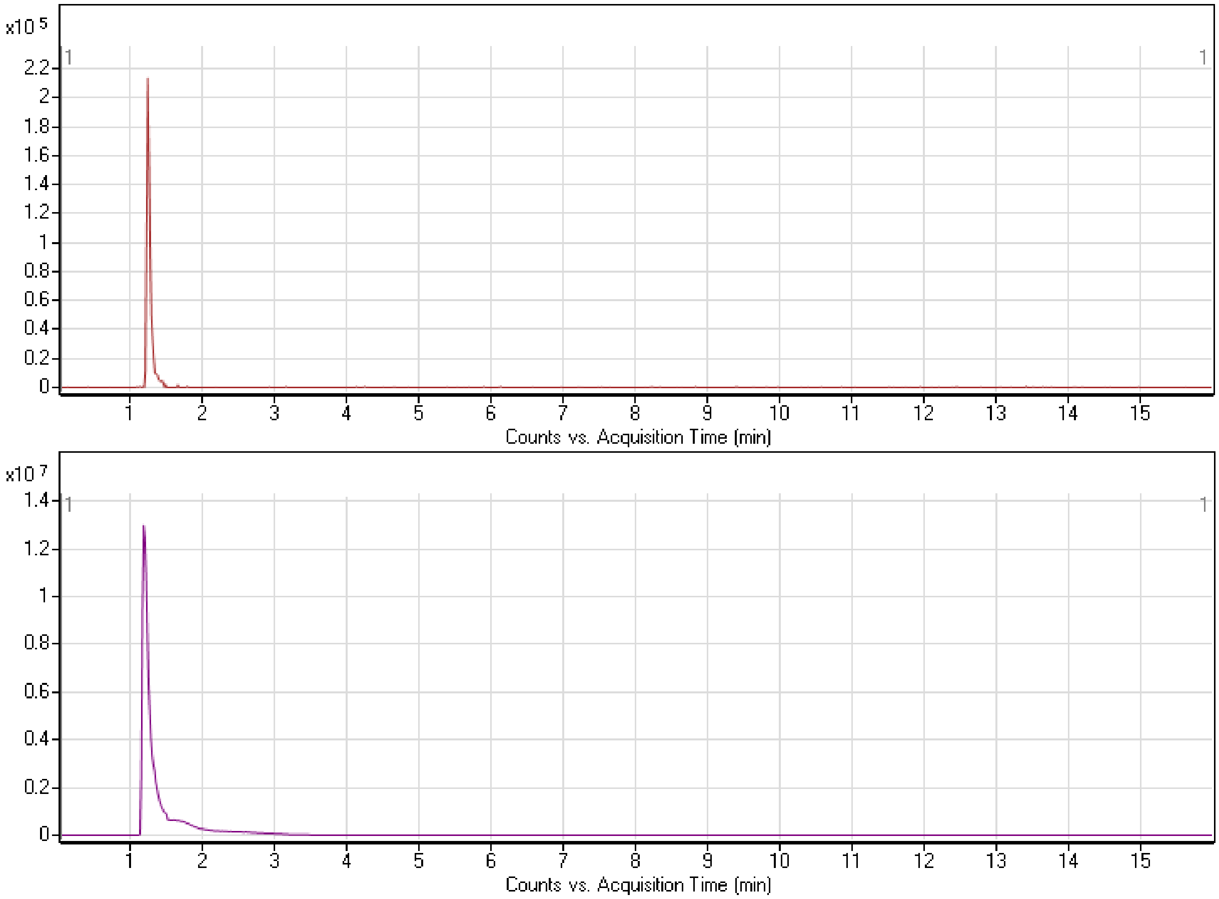

Identification of hippurate was confirmed by mathcing m/z and retention times of m/z 180 in plasma sample (upper panel) with synthetic standard of hippurate (lower panel).

Supplementary Figure S9. Annotation of Homocitrulline

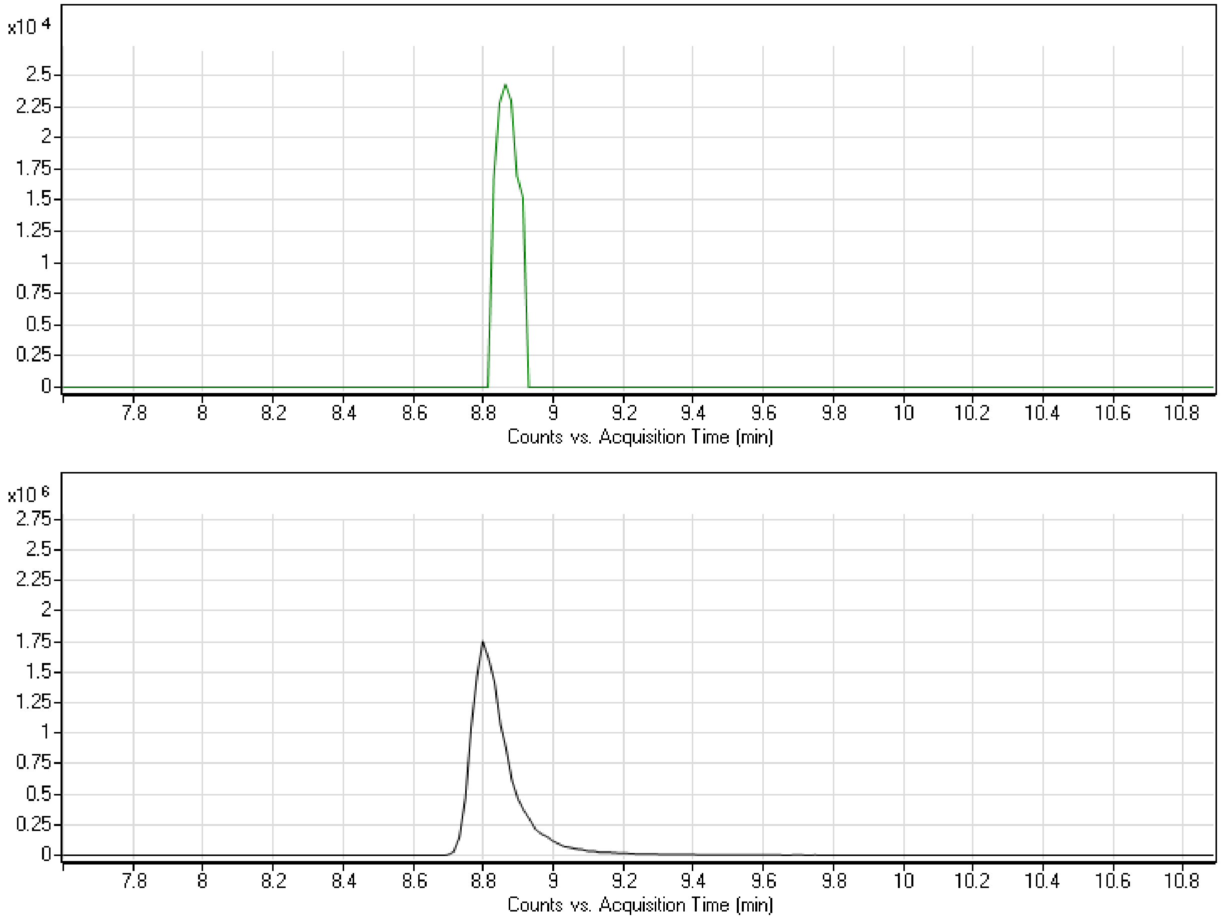

Identification of homocitrulline was confirmed by mathcing m/z and retention times of m/z 190 in plasma sample (upper panel) with synthetic standard of homoctirulline (lower panel).

Supplementary Figure S10. Annotation of Lysine

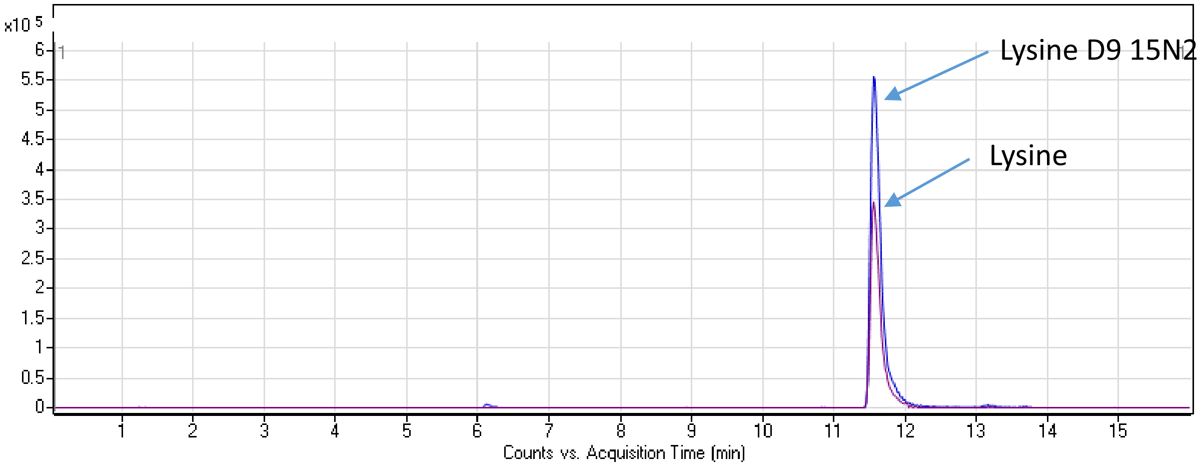

Identification of lysine was confirmed by mathcing chromatographic retention time with isotope-labeled internal standard (Lysine D9 15N2).

Supplementary Figure S11. Annotation of Tryptophan

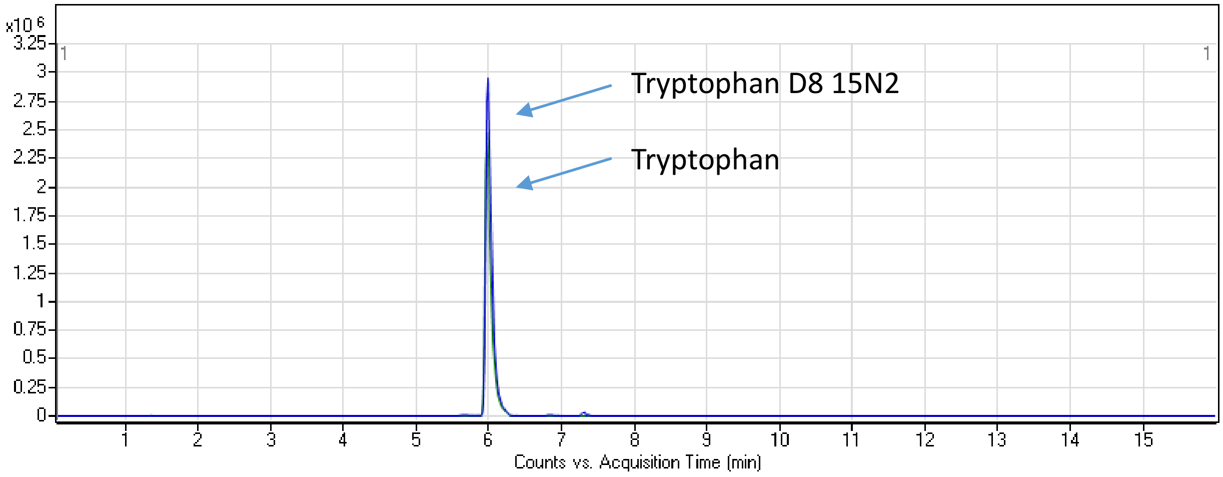

Identification of tryptophan was confirmed by mathcing chromatographic retention time with isotope-labeled internal standard (Tryptophan D8 15N2).

Supplementary Figure S12. Annotation of Threonine

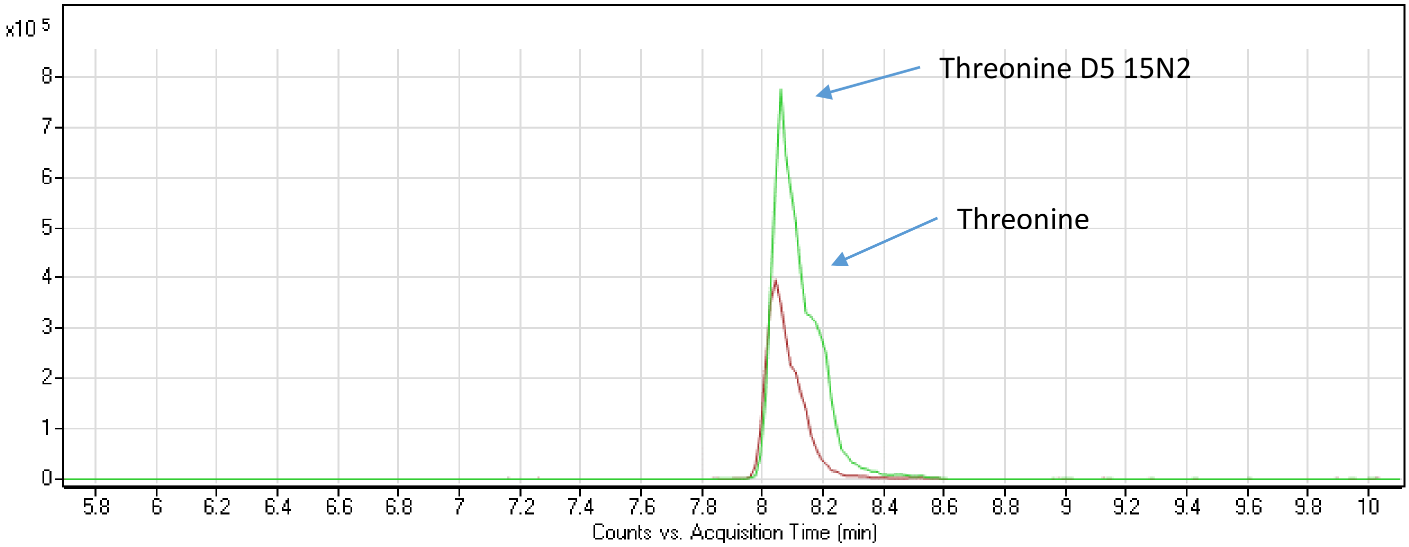

Identification of threonine was confirmed by mathcing chromatographic retention time with isotope-labeled internal standard (Threonine D5 15N2).

References

1. Ottosson, F., et al., *Dimethylguanidino Valerate: A Lifestyle-Related Metabolite Associated With Future Coronary Artery Disease and Cardiovascular Mortality.* J Am Heart Assoc, 2019. **8**(19): p. e012846.
2. O'Sullivan, J.F., et al., *Dimethylguanidino valeric acid is a marker of liver fat and predicts diabetes.* J Clin Invest, 2017.
